# Analgesic medicines for adults with low back pain: protocol for a systematic review and network meta-analysis

**DOI:** 10.1186/s13643-020-01506-3

**Published:** 2020-11-04

**Authors:** Michael A. Wewege, Matthew K. Bagg, Matthew D. Jones, James H. McAuley, The ANiMALIA investigators, Aidan G. Cashin, Richard O. Day, Michael C. Ferraro, Thiago Folly, Sylvia M. Gustin, Amanda D. Hagstrom, Hayley B. Leake, Colleen K. Loo, Andrew J. McLachlan, Edel T. O’Hagan, Rodrigo R. N. Rizzo, Siobhan M. Schabrun

**Affiliations:** 1 School of Medical Sciences, Faculty of Medicine, University of New South Wales, Sydney, Australia; 2 Centre for Pain IMPACT, Neuroscience Research Australia, Sydney, Australia; 3 Prince of Wales Clinical School, University of New South Wales, Sydney, NSW 2052 Australia; 4 New College Village, University of New South Wales, Sydney, Australia

**Keywords:** Analgesics [MeSH], Low back pain [MeSH], Meta-analysis as topic [MeSH], Network meta-analysis [MeSH], Systematic reviews as topic [MeSH]

## Abstract

**Background:**

There is limited evidence for the comparative effectiveness of analgesic medicines for adults with low back pain. This systematic review and network meta-analysis aims to determine the analgesic effect, safety, acceptability, effect on function, and relative rank according to analgesic effect, safety, acceptability, and effect on function of a single course of [an] analgesic medicine(s) or combination of these medicines for people with low back pain.

**Methods:**

We will include published and unpublished randomised trials written in any language that compare an analgesic medicine to either another medicine, placebo/sham, or no intervention in adults with low back pain, grouped according to pain duration: acute (fewer than 6 weeks), sub-acute (6 to 12 weeks), and chronic (greater than 12 weeks). The co-primary outcomes are pain intensity following treatment and safety (adverse events). The secondary outcomes are function and acceptability (all-cause dropouts). We will perform a network meta-analysis to compare and rank analgesic medicines. We will form judgements of confidence in the results using the Confidence in Network Meta-Analysis (CINeMA) methodology.

**Discussion:**

This network meta-analysis will establish which medicine, or combination of medicines, is most effective for reducing pain and safest for adults with low back pain.

**Systematic review registration:**

PROSPERO CRD42019145257

**Supplementary Information:**

The online version contains supplementary material available at 10.1186/s13643-020-01506-3.

## Background

Low back pain (LBP) is a global health problem; it is the leading cause of disability in 126 out of 195 countries according to the Global Burden of Disease Study 2017 [1]. Non-specific LBP represents over 90% of cases, in which a pathoanatomical source of pain cannot be reliably identified [2, 3], and is classified by the duration of pain: acute (fewer than 6 weeks), sub-acute (6 to 12 weeks), and chronic (greater than 12 weeks) [4, 5]. The estimated lifetime prevalence of LBP is up to 80%, meaning many adults will experience an episode of LBP at least once [6, 7]. Following onset, most people experience a reduction in pain within the first 6 weeks [8]. However, only 60% are fully recovered at 12 weeks and 70% will experience a recurrence of LBP within 12 months of recovery [9, 10]. Approximately 20% of the global population live with chronic LBP at any time [7].

Despite recommendations for non-pharmacological interventions to play a greater role in the management of LBP [11], analgesic medicines are the most commonly prescribed intervention across a range of primary care settings (e.g. general practice, emergency department) [12–18]. Analgesic medicines include non-steroidal anti-inflammatory drugs (NSAIDs), opioids, paracetamol (acetaminophen), anti-convulsants, anti-depressants, muscle relaxants, and corticosteroids. These medicines are used in a range of countries [13–16, 19, 20], but there appear to be no common patterns to prescribing [20]. For example, muscle relaxant medicines are commonly prescribed in the USA and Italy [14, 15, 19], but seldom in Australia [12].

People with LBP (and clinicians) want to know the most effective medicine for their condition [21–24]. This requires information about comparative effectiveness—the effect of a medicine compared to other medicines—to be available for clinical decision making. Comparative effectiveness has been insufficiently described in syntheses of the literature to date [25–35]. This is understandable, as most of these systematic reviews [28, 29, 31, 32] investigated a single comparison, usually efficacy, the effect of a medicine compared to sham. Several of these reviews [25, 35] additionally examined a limited number of effectiveness comparisons. No quantitative synthesis was made of these data, which was appropriate because methods for single comparisons should not be used across multiple comparisons. Comparative effectiveness data that are limited and lack synthesis have low utility for clinical decision-making.

Network meta-analysis (NMA) can be used to synthesise data across multiple comparisons. NMA provides valid estimates of comparative effectiveness by fitting a single statistical model to a connected network of interventions when there is confidence in the assumptions of transitivity and coherence (see the ‘Assumption of transitivity’ and ‘Assessment of network heterogeneity and coherence’ sections) [36–39]. The results are relative effect estimates for each comparison between medicines of interest. These data are applicable to decision scenarios if adults with LBP, clinicians, and policy makers are determining *which* of several medicines should be used. The relative effect estimates can be used to rank interventions based on their effect on relevant outcomes, which may also assist clinical decision-making. Therefore, this NMA will evaluate the comparative effectiveness of analgesic medicines for adults with LBP.

### Objectives

The objectives of this review are to:

1. Determine the effect on pain intensity, safety, acceptability, and effect on function of a single course of [an] analgesic medicine(s) or combination of these medicines.
2. Determine a relative rank for each intervention according to its effect on pain intensity, safety, acceptability, and effect on function.

## Methods

This protocol follows the Preferred Reporting Items for Systematic Reviews and Meta-Analysis Protocols (PRISMA-P) guidelines [40, 41], provided as Additional file 1. The review was registered with the International Prospective Register of Systematic Reviews on 31 October 2019 (PROSPERO ID: CRD42019145257).

### Eligibility criteria for inclusion in the review

#### Study design

We will include randomised trials (RCTs) that provide at least one comparison between two interventions of interest (see the ‘Interventions’ section). There are no restrictions on language or publication status—we will include unpublished data because it may meaningfully alter the relative effectiveness of a medicine [42, 43]. We will include data from parallel group trials. We will also include data from the first phase of crossover RCTs because of the lack of relative stability in recent-onset LBP and known carry over effects in different classes of analgesic medicines (e.g. anti-depressants) [44, 45]. This approach has been used in published NMAs [36, 46, 47]. We will exclude cluster RCTs.

We will exclude enriched-enrolment RCTs. Although the single-arm run-in phase of enriched-enrolment trials might provide information on adverse effects, the NMA will not include data from uncontrolled trials. Additionally, the double-blind/randomised phase of enriched-enrolment RCTs has questionable external validity [48] and is limited in detecting adverse effects [48, 49].

#### Participants

We will include studies that randomised adults with non-specific LBP, defined as a primary area of pain between the twelfth rib and gluteal fold, with or without associated leg pain [4, 5]. We will consider three different pain durations, which we will analyse separately: acute (fewer than 6 weeks), sub-acute (6 to 12 weeks), and chronic (greater than 12 weeks) [4]. We will include studies that randomised participants with heterogeneous pain conditions if separate data is obtainable for the participants with LBP. Participants may be experienced or naïve to the trial intervention, which we will assess for the evaluation of transitivity (see the ‘Assumption of transitivity’ section). We will exclude interventional (surgical/operative) settings and studies where greater than 20% of participants have leg pain that meets the definition of sciatica according to Koes et al. [50], where patients have unilateral leg pain greater than LBP radiating to the foot or toes with associated neurological indications, or where LBP is attributable to a specific pathology, such as infection, neoplasm, metastasis, inflammatory disease, or fractures.

#### Interventions

We will include medicines from the following classes: NSAIDs, paracetamol (acetaminophen), opioid analgesics, anti-convulsants, anti-depressants, muscle relaxant medicines, and corticosteroids. These medicines must be listed on the World Health Organization (WHO) Anatomical Therapeutic Chemical (ATC) system and licensed for current use by at least one of the following agencies: US Food and Drug Administration (FDA), the Australian Therapeutic Goods Administration (TGA), the European Medicines Agency (EMA), or the Medicine and Healthcare Products Regulatory Agency (MHRA). These interventions of interest are listed in Additional file 2 with their ATC codes and licensing status. We will include additional analgesic medicines from these classes, identified during the review, provided they are licensed by one of the above agencies. Medicines may be delivered as mono or combination therapy via any systemic route of administration (e.g. oral, intravenous, intramuscular, buccal, sublingual). We will exclude non-systemic administration (such as topical, intraarticular, or epidural administration). We will not exclude studies that assign non-pharmacological co-interventions to one or more of the intervention arms. We will consider these studies in the evaluation of transitivity [36].

We will represent each intervention of interest as a separate node in a network of all possible comparisons between interventions (not shown due to visual complexity). We define the placebo/sham intervention node as any drug intervention that does not contain an active analgesic ingredient (including ‘active’ placebo arms). We consider that no-treatment includes continuation of usual care or being placed on a waitlist. We will further define nodes according to route of administration, which means that a single drug may be represented by multiple nodes in the network (e.g. oral diclofenac and intravenous diclofenac are separate nodes).

We will classify the dose of each medicine as either (i) standard dosing range (SDR), (ii) below the SDR, or (iii) above the SDR. We will source the relevant SDR for each medicine using the following hierarchy: Prescriber’s Digital Reference [51], MIMS [52], or the Australian Medicines Handbook [53]. If the SDR is not provided by any of these sources, we will use the medicine’s licensed dosing range (LDR). Typically, the LDR is equivalent to the SDR used in clinical practice, although the SDR may be lower than the LDR. We will identify a medicine’s LDR using the following hierarchy: FDA, MHRA, EMA, or TGA. Where a study contains two or more intervention arms within the same dosing range, we will combine these arms using formulae in the *Cochrane Handbook for Systematic Reviews of Interventions* [54].

### Outcomes

The primary outcomes for this review are pain and safety.

- Pain is defined as pain intensity, measured at the time point closest to the end of treatment. Pain intensity may be measured with a continuous self-report scale (e.g. visual analogue scale (VAS) or numeric rating scale (NRS)), a rating scale within a composite measure of pain (e.g. McGill Pain Questionnaire), or an ordinal scale (we will consider such ordinal scales to exhibit continuous properties). We will not exclude studies that use other measurement tools.
- Safety is the number of participants who experience an adverse effect during the treatment period. We define adverse effects according to the US FDA, as ‘any untoward medical occurrence associated with the use of a drug in humans, whether or not considered drug related’ is considered an adverse effect (55). We will consider ‘adverse event’, ‘adverse drug reaction’, ‘side effect’, ‘toxic effect’, or ‘complication’ as indicating adverse effect. No change or an increase in pain intensity is not considered an adverse effect.

The secondary outcomes are function, serious adverse events, and acceptability:

- Function, defined as low back specific function, measured at the end of treatment, may be measured with a continuous, self-report scale (e.g. Roland Morris Disability Questionnaire (RMDQ) or Oswestry Disability Index (ODI)), a rating scale within a composite measure (e.g. Short Form-36), or an ordinal scale (we will consider such ordinal scales to exhibit continuous properties). We will not exclude studies that use other measurement tools.
- We will also compare the number of participants who experience a serious adverse effect during the treatment period using the US FDA classification [55, 56], where a serious adverse effect: ‘results in death; is life threatening; requires inpatient hospitalisation or causes prolongation of existing hospitalisation; results in persistent or significant disability/incapacity; may have caused a congenital anomaly/birth defect; or requires intervention to prevent permanent impairment or damage’. A consistent definition of serious adverse effect ensures the different medicines can be compared across the network.
- Acceptability is defined as the number of participants who leave the trial for any reason before the end of treatment [47].

### Search strategy and study selection

We will search the following electronic databases from inception to current:

- MEDLINE (Ovid) (1946 to current)
- EMBASE (Ovid) (1980 to current)
- CINAHL (EBSCO) (1982 to current)
- Cochrane Central Register of Controlled Trials (CENTRAL) in the Cochrane Library, current issue
- ClinicalTrials.gov (ClinicalTrials.gov/ct2/home)
- EU Clinical Trials Register (eudract.ema.europa.eu)
- WHO International Clinical Trial Registry Platform (apps.who.int/trialsearch/Default.aspx)

Our search strategies incorporate the recommended strategies from the Cochrane Back and Neck Group (4) to identify randomised trials of low back pain and terms for the interventions of interest [36]. The search strategy for MEDLINE is listed in Additional file 3. We will also search previous systematic reviews and the reference lists of included studies to identify any additional trials. Records identified through all searches will be downloaded and managed in a custom relational database.

We will conduct record screening in Covidence systematic review software [57]. We will conduct two stages of screening: (i) title and abstract and (ii) full text. Two reviewers will independently screen studies for eligibility at each stage. Disagreements will be resolved through discussion, with arbitration from a third author (JHM) if required. We will contact a study’s corresponding author up to three times to obtain additional information to determine eligibility, and if no reply is received, we will exclude the study from this iteration of the review. Studies in languages other than English will be translated. We will summarise the literature search using an adapted PRISMA flow diagram [58].

### Record management

We will manage the included records in the relational database. We will conduct record linkage to establish unique studies for data extraction, which may consist of multiple records. We will search for the protocols and trial registrations of all included trials. We will use the following hierarchy to prioritise records for data extraction: (i) primary report (typically the journal article reporting the results of the primary analysis of the trial), (ii) secondary report (secondary analysis of the trial), (iii) conference abstract (a report of a secondary analysis), (iv) trial registration, (v) other secondary records, and (vi) other conference abstracts.

### Data extraction

Two reviewers will independently extract and enter data from included trials into standardised spreadsheets. Review authors will not extract data from any trial in which they have had any involvement. Data will be taken from previous reviews conducted by the authors when possible. Disagreements between reviewers will be resolved through discussion, with arbitration from a third author (JHM) if required. We will not extract data from interventions that do not meet the eligibility criteria for this review.

We will extract data on:

*Trial characteristics*: country, setting, and number of trial sites; sample size; and study duration.

*Participants:* diagnosis, duration of LBP, age, male/female ratio, arm-level pain intensity at baseline (as mean (standard deviation [SD])), experience or naivety with the trial intervention, and co-morbidities, including alternate sites of pain.

*Interventions*: medicine(s) tested, control; duration of intervention; dosage regimen; routes of administration; and usage of rescue medication.

*Outcomes*: type and dimensions of the scale/measure used to assess pain or function and the time from randomisation at which the end of treatment data were obtained in individual trials. We will extract the definition of ‘adverse effect’ and ‘serious adverse effect’ used in each study. We will extract data on study results including participant allocation to each intervention group; compliance to the intervention (including the definition of compliance); the number of participants who discontinued due to an adverse event; the event rate and descriptions of all reported adverse effects; and pain intensity and function at the completion of treatment.

If studies report more than one measure for pain, we will prioritise extraction in the following order: 100 mm VAS, 10 cm VAS, 11-point NRS, rating scale for pain intensity from a composite measure of pain (e.g. McGill Pain Questionnaire), and ordinal scale. We will preferentially extract the outcome score and measure of variance at the end of treatment (or closest time point) for each group, followed by the change from baseline and measure of variance. If data are not available for each trial arm, we will extract the between-group statistics at the end of treatment.

If studies report more than one measure for function, we will prioritise extraction in the following order: ODI, RMDQ, rating scale for functional ability from a composite measure, and ordinal scale. We will preferentially extract the outcome score and measure of variance at the end of treatment (or closest time point) for each group, followed by the change from baseline and measure of variance. If data are not available for each trial arm, we will extract the between-group statistics at the end of treatment.

### Missing data

We will contact a trial’s corresponding author up to three times via email to request missing data, which will be considered unobtainable if no reply is received within 6 weeks. If data for outcomes of pain and function are not presented in an appropriate form for meta-analysis (such as median and range instead of SDs, standard errors, *t*-statistics, or *p* values), we will attempt to impute these using established methods [54, 59]. We will conduct sensitivity analyses for pain at the end of treatment and safety if we impute missing data for either of these outcomes.

### Risk of bias

We will appraise each trial’s risk of bias using the Cochrane ‘Risk of bias’ tool, version 5.1 [54] and recommendations by Furlan et al. [4]. Two reviewers will independently appraise trial-level risk of bias for 13 items across the domains of selection, performance, attrition, detection, reporting, and other sources of bias. If an item is typically rated at outcome level, which may differ between our two primary outcomes (pain intensity and safety), we will use the more conservative rating (e.g. using high risk over unclear risk). Review authors will not appraise risk of bias for any trial in which they have had any involvement (e.g. trial investigator). Risk of bias assessments will be taken from previous reviews of analgesic medicines conducted by our author team, where the same approach was used.

We will determine an overall risk of bias for each trial by adapting the process from Furukawa et al. [47]: low overall risk is determined when three or fewer items are rated ‘unclear’ risk and no domains are rated ‘high’; moderate overall risk is determined if a single item is rated as ‘high’ risk of bias, or no item is rated as ‘high’ risk but four or more are rated as ‘unclear’; and high overall risk otherwise.

## Data synthesis

We will perform separate analyses for the three classifications of pain duration: acute (fewer than 6 weeks), sub-acute (6 to 12 weeks), and chronic (greater than 12 weeks).

### Summary of the network

Within each classification, we will present descriptive statistics for each included trial, including the comparison(s) and a clinical/methodological summary (e.g. year of publication, sponsorship, clinical setting). We will represent the network of trials in a network graph; the size of the node will reflect the total number of participants, the width of each edge will reflect the number of studies presenting direct evidence for the comparison, and the colour of each edge will represent the overall risk of bias (see the ‘Risk of bias’ section).

### Pairwise comparisons

We will synthesise the data for each comparison using pairwise random-effects meta-analysis in R [60]. We will compare the effects of competing interventions on pain and function using mean differences (MD) with 95% confidence intervals (CIs) and on safety and acceptability using odds ratios (OR) with 95% CIs. For pain intensity and function, we will convert outcome data to common 0- to 100-point scales (mean (SD)), which has been used in reviews of analgesic medicines to enable greater clinical translation of results [25, 29, 33, 36]. This approach is based on evidence that measurement scales within the constructs of pain intensity and function are highly correlated [61, 62] and enables different types of study data to be pooled (e.g. endpoint, change from baseline).

We assume that the heterogeneity variance is different for each comparison in pairwise meta-analyses and will estimate this parameter (*τ*^2^) for each comparison. We will also test for the presence of statistical heterogeneity within each comparison using the *Q* statistic. We will calculate 95% prediction intervals and consider intervals spanning greater than 15 points (on a 0- to 100-point scale) on either side to indicate important heterogeneity [36]. We will visually inspect the distribution of effect sizes in the forest plots for each comparison and consider an *I*^2^ value greater than 50% indicative of important variability across studies that is not due to sampling error [63, 64].

### Assumption of transitivity

Transitivity is the key assumption underlying the valid estimation of effects for indirect comparisons in NMA [38, 39]. Transitivity is the assumption that the distributions of effect modifiers (covariates associated with intervention effects) are balanced across comparisons in the network [39, 65]. Given the lack of evidence for robust effect modifiers in LBP trials [66], we have used clinical and methodological experience to identify the following potential effect modifiers:

- Baseline mean pain intensity (continuous variable)
- Assigned co-interventions (dichotomous variable), categorised as (i) yes or (ii) no
- Small sample size [67] (dichotomous variable, categorised as (i) total sample fewer than 50 participants and (ii) total sample greater than 50 participants)
- Experience with test medicine (dichotomous variable), categorised as (i) yes or (ii) no
- Naivety to test medicine (dichotomous variable), categorised as (i) yes or (ii) no
- Dose of medicine (trichotomous variable), categorised as (i) SDR, (ii) below the SDR, or (iii) above the SDR

We will represent the distribution of these effect modifiers in a range of covariate contribution plots [68]. The authors will visually assess the distributions of effect modifiers across all treatment comparisons in the network and determine by consensus whether there is sufficient dissimilarity between comparisons in the network to threaten the assumption of transitivity. We will explore the influence of effect modifiers that demonstrate dissimilarity on incoherence/heterogeneity using network meta-regression or subgroup analyses (or both). We will consider not proceeding with NMA, or altering the network structure, if we observe considerable dissimilarity. We anticipate that insufficient reporting of effect modifiers and pairwise comparisons containing few studies will limit the assessment of transitivity [38].

### Network meta-analysis

We will perform random-effects NMA within an electrical network and graph theory framework using the *netmeta* package in R [69, 70]. We will account for multi-arm trials using the weighting method based on back-calculating variances (using the Laplacian matrix and its pseudoinverse) [70]. We will use MDs on a common 0 to 100-point scale for pain and function and ORs for safety and acceptability. We will present the results for each intervention compared to placebo in NMA forest plots for each outcome. We will consider a 10-point difference to constitute the minimal clinically important difference for pain intensity and function [26].

We will rank the effect of all interventions on pain intensity and safety using *P*-scores, a method for estimating treatment rankings that does not require re-sampling methods [71]. We will present a contributions matrix to indicate the weighting of direct evidence contributions to each NMA effect size, which will also be used to evaluate the confidence in the overall evidence (see the ‘Confidence in cumulative evidence’ section) [72]. We will illustrate the contribution of each design to an NMA effect size using net heat plots [73].

### Assessment of network heterogeneity and coherence

We will assume a common heterogeneity variance across the network [74]. We will present the estimate for this parameter (*τ*^2^_network_) from the NMA models and the estimated proportion of variability across the entire network that is not due to sampling error (*I*^2^_network_). We will estimate the *Q* statistics for total network heterogeneity (*Q*_total_), heterogeneity within designs (*Q*_within_), and heterogeneity between designs (*Q*_between_), where designs constitute the individual elements of the set of trial designs.

Coherence is a property of closed loops of evidence, whereby it reflects the agreement between direct and indirect treatment effects [38]. We will evaluate coherence across the entire network using the *Q* statistics (above); the decomposed *Q*_within_ and *Q*_between_, an alternative estimate for *Q*_between_ using the ‘design-by-treatment’ interaction model [75, 76]; and the Separating Indirect from Direct Evidence (SIDE; aka node-splitting) approach [77]. We will illustrate the extent of incoherence across the network using net heat plots [73]. We will illustrate local coherence estimates using forest plots grouped into direct and indirect estimates for each available comparison. We will form judgements about important incoherence using all the measures of global and local heterogeneity and coherence.

If we encounter important incoherence, we will examine the dataset for data extraction errors and explore the observed incoherence using pre-specified covariates in network meta-regression and subgroup analyses, provided sufficient studies are available. We may consider not proceeding with NMA if important unexplained incoherence remains. This judgement will involve the clinical and methodological evaluation of transitivity, the approaches to identify incoherence, and the knowledge that small amounts of incoherence may be due to chance [78].

### Meta-regression

We aim to perform random-effects network meta-regression within a Bayesian hierarchical framework using the *gemtc* package in R [79]. The *gemtc* package will automatically determine uninformative prior distributions for all parameters in our model [80], which are commonly applied in NMA [47, 81]. We will run the Markov Chain Monte Carlo simulation with four chains for each model, using 100,000 iterations, a burn-in of 5000 iterations and extraction of every 10th value. We will assess convergence with the Gelman-Rubin-Brooks plot and Potential Scale Reduction Factor (a threshold of < 1.05 indicates adequate convergence).

We will investigate baseline pain intensity (continuous variable) and sample size (dichotomous variable) as possible sources of incoherence or heterogeneity by default. We will specify our assumptions (common or exchangeable covariate-comparison interaction) for each network meta-regression model once the data are available, so as to make best use of the available data [82–84]. We assume coherent relative treatment effects estimated at the covariate value 0 and coherent regression coefficients for the treatment effect by covariate interaction [84, 85]. We hypothesise that:

- Increasing baseline pain intensity increases the effect size between intervention and comparator.
- Increasing sample size reduces the effect size between intervention and comparator.

We may also investigate clinical or methodological factors identified during the review process that may threaten transitivity as sources of incoherence or heterogeneity, or both [86].

### Sensitivity analysis

We will conduct sensitivity analyses on pain and safety excluding studies at high risk of bias, provided the original network structure remains the same. We will also conduct sensitivity analyses on pain and safety excluding doses above or below the SDR, provided the original network structure remains the same. If sufficient data are not available for network meta-regression baseline pain intensity or sample size, we will conduct sensitivity analyses by removing trials with baseline mean pain intensity higher than 70/100 (VAS) or sample size less than 50, respectively. We will also conduct sensitivity analyses for pain at end of treatment and safety if we impute missing data for either of these outcomes. We will consider the effects in the sensitivity analyses to be important when their interpretation differs compared to the primary analysis, for example, if a statistically significant effect becomes non-significant.

### Meta-bias(es)

We will assess small study effects in pairwise comparisons using comparison-adjusted [82] and contour-enhanced [87] funnel plots when there are at least 10 studies available. Such plots assist interpretation of asymmetry that is due to publication bias rather than other factors, such as lesser methodological quality. We will interpret an absence of studies in areas of non-significance as suggestive of publication bias for that pairwise comparison.

## Confidence in cumulative evidence

We will form judgements of confidence in the effect estimates and rankings for pain, safety, function, and acceptability using the Confidence in Network Meta-Analysis (CINeMA) web application [88, 89]. CINeMA considers six domains: within-study bias, reporting bias, indirectness, imprecision, heterogeneity, and incoherence (Additional file 4) [72]. Initially, judgements will be rated as ‘high’ because all included trials will be RCTs.

### Summary of findings

We will present the results of the NMA in adapted ‘Summary of findings’ tables for pain and safety [90, 91]. The tables will contain details of the clinical question, a network geometry plot, relative and absolute effect estimates, certainty of evidence, ranking of treatments, and interpretation of findings.

## Supplementary Information

**Additional file 1.** PRISMA-P checklist.

**Additional file 2.** Interventions of Interest.

**Additional file 3.** MEDLINE search strategies.

**Additional file 4.** CINeMA.

## Data Availability

Data sharing is not applicable to this article as no datasets were generated or analysed during the current study.

## Abbreviations

ATC: Anatomical Therapeutic Chemical
CINeMA: Confidence in Network Meta-Analysis
CI: Confidence interval
EMA: European Medicines Agency
LDR: Licensed dosing range
LBP: Low back pain
MD: Mean difference
MHRA: Medicine and Healthcare Products Regulatory Agency
NSAID: Non-steroidal anti-inflammatory drug
NMA: Network meta-analysis
NRS: Numeric rating scale
OR: Odds ratio
ODI: Oswestry Disability Index
PRISMA: Preferred Reporting Items for Systematic Reviews and Meta-Analysis
RCT: Randomised trial
RMDQ: Roland Morris Disability Questionnaire
SD: Standard deviation
SDR: Standard dosing range
TGA: Therapeutic Goods Administration
FDA: US Food and Drug Administration
VAS: Visual analogue scale
WHO: World Health Organization

## References

[1] James SLAD, Abate KH, Abay SM, Abbafati C, Abbasi N (2018). Global, regional, and national incidence, prevalence, and years lived with disability for 354 diseases and injuries for 195 countries and territories, 1990-2017: a systematic analysis for the Global Burden of Disease Study 2017. Lancet..

[2] Koes BW, van Tulder MW, Thomas S. Diagnosis and treatment of low back pain. BMJ. 2006;332(7555):1430–4. 10.1136/bmj.332.7555.1430 PMC1479671 16777886

[3] Maher C, Underwood M, Buchbinder R. Non-specific low back pain. Lancet. 2017;389(10070):736–47. 10.1016/S0140-6736(16)30970-9 27745712

[4] Furlan AD, Malmivaara A, Chou R, Maher CG, Deyo RA, Schoene M (2015). 2015 Updated Method Guideline for Systematic Reviews in the Cochrane Back and Neck Group. Spine..

[5] Treede R-D, Rief W, Barke A, Aziz Q, Bennett MI, Benoliel R (2015). A classification of chronic pain for ICD-11. Pain..

[6] Rubin DI (2007). Epidemiology and risk factors for spine pain. Neurol Clin..

[7] Hoy D, Bain C, Williams G, March L, Brooks P, Blyth F, et al. A systematic review of the global prevalence of low back pain. Arthritis Rheumatol. 2012;64(6):2028–37. 10.1002/art.34347 22231424

[8] da C Menezes Costa L, Maher CG, Hancock MJ, McAuley JH, Herbert RD, Costa LOP. The prognosis of acute and persistent low-back pain: a meta-analysis. Can Med Assoc J. 2012;184(11):E613–E24. 10.1503/cmaj.111271 PMC3414626 22586331

[9] Henschke N, Maher CG, Refshauge KM, Herbert RD, Cumming RG, Bleasel J (2008). Prognosis in patients with recent onset low back pain in Australian primary care: inception cohort study. BMJ..

[10] da Silva T, Mills K, Brown BT, Pocovi N, de Campos T, Maher C, et al. Recurrence of low back pain is common: a prospective inception cohort study. J Physiother. 2019;65(3):159–65. 10.1016/j.jphys.2019.04.010 31208917

[11] Oliveira CB, Maher CG, Pinto RZ, Traeger AC, Lin C-WC, Chenot J-F, et al. Clinical practice guidelines for the management of non-specific low back pain in primary care: an updated overview. Eur Spine J. 2018;27(11):2791–803. 10.1007/s00586-018-5673-2 29971708

[12] Mathieson S, Valenti L, Maher CG, Britt H, Li Q, McLachlan AJ (2018). Worsening trends in analgesics recommended for spinal pain in primary care. Eur Spine J..

[13] Bishop PB, Wing PC (2003). Compliance with clinical practice guidelines in family physicians managing worker's compensation board patients with acute lower back pain. Spine J..

[14] Ivanova JI, Birnbaum HG, Schiller M, Kantor E, Johnstone BM, Swindle RW. Real-world practice patterns, health-care utilization, and costs in patients with low back pain: the long road to guideline-concordant care. Spine J. 2011;11(7):622–32. 10.1016/j.spinee.2011.03.017 21601533

[15] Piccoliori G, Engl A, Gatterer D, Sessa E, in der Schmitten J, Abholz HH. Management of low back pain in general practice - is it of acceptable quality: an observational study among 25 general practices in South Tyrol (Italy). BMC Fam Pract. 2013;14:148. 10.1186/1471-2296-14-148 PMC3850994 24090155

[16] Williams CM, Maher CG, Hancock MJ, McAuley JH, McLachlan AJ, Britt H, et al. Low back pain and best practice care: a survey of general practice physicians. Arch Intern Med. 2010;170(3):271–7. 10.1001/archinternmed.2009.507 20142573

[17] Ferreira GE, Machado GC, Abdel Shaheed C, Lin C-WC, Needs C, Edwards J (2019). Management of low back pain in Australian emergency departments. BMJ Qual Saf.

[18] Friedman BW, Chilstrom M, Bijur PE, Gallagher EJ (2010). Diagnostic testing and treatment of low back pain in United States emergency departments: a national perspective. Spine..

[19] Gore M, Tai KS, Sadosky A, Leslie D, Stacey BR (2012). Use and costs of prescription medications and alternative treatments in patients with osteoarthritis and chronic low back pain in community-based settings. Pain Pract..

[20] Hart OR, Uden RM, McMullan JE, Ritchie MS, Williams TD, Smith BH (2015). A study of National Health Service management of chronic osteoarthritis and low back pain. Prim Health Care Res Dev..

[21] Del Fiol G, Workman TE, Gorman PN. Clinical questions raised by clinicians at the point of care: a systematic review. JAMA Int Med. 2014;174(5):710–8. 10.1001/jamainternmed.2014.368 24663331

[22] Chou L, Ranger TA, Peiris W, Cicuttini FM, Urquhart DM, Sullivan K, et al. Patients’ perceived needs of health care providers for low back pain management: a systematic scoping review. Spine J. 2018;18(4):691–711. 10.1016/j.spinee.2018.01.006 29373836

[23] Chou L, Ranger TA, Peiris W, Cicuttini FM, Urquhart DM, Sullivan K, et al. Patients’ perceived needs for medical services for non-specific low back pain: a systematic scoping review. PLoS One. 2018;13(11):e0204885. 10.1371/journal.pone.0204885 PMC6224057 30408039

[24] Lim YZ, Chou L, Au RTM, Seneviwickrama KLMD, Cicuttini FM, Briggs AM, et al. People with low back pain want clear, consistent and personalised information on prognosis, treatment options and self-management strategies: a systematic review. J Physiother. 2019;65(3):124–35. 10.1016/j.jphys.2019.05.010 31227280

[25] Abdel Shaheed C, Maher CG, Williams KA, McLachlan AJ. Efficacy and tolerability of muscle relaxants for low back pain: systematic review and meta-analysis. Eur J Pain. 2017;21(2):228–37. 10.1002/ejp.907 27329976

[26] Chou R, Deyo R, Friedly J, Skelly A, Weimer M, Fu R, et al. Systemic pharmacologic therapies for low back pain: a systematic review for an American College of Physicians clinical practice guideline. Ann Int Med. 2017;166(7):480–92. 10.7326/M16-2458 28192790

[27] Deyo RA, Von Korff M, Duhrkoop D. Opioids for low back pain. BMJ. 2015;350:g6380. 10.1136/bmj.g6380 PMC6882374 25561513

[28] Enke O, New HA, New CH, Mathieson S, McLachlan AJ, Latimer J, et al. Anticonvulsants in the treatment of low back pain and lumbar radicular pain: a systematic review and meta-analysis. Can Med Assoc J. 2018;190(26):E786–E93. 10.1503/cmaj.171333 PMC6028270 29970367

[29] Machado GC, Maher CG, Ferreira PH, Day RO, Pinheiro MB, Ferreira ML. Non-steroidal anti-inflammatory drugs for spinal pain: a systematic review and meta-analysis. Ann Rheum Dis. 2017;76(7):1269–78. 10.1136/annrheumdis-2016-210597 28153830

[30] Qaseem A, Wilt TJ, McLean RM, Forciea MA (2017). Noninvasive treatments for acute, subacute, and chronic low back pain: a clinical practice guideline from the American College of Physicians. Ann Intern Med..

[31] Saragiotto BT, Machado GC, Ferreira ML, Pinheiro MB, Abdel Shaheed C, Maher CG. Paracetamol for low back pain. Cochrane Database Syst Rev. 2016(6):CD012230. 10.1002/14651858.CD012230 PMC6353046 27271789

[32] Urquhart DM, Hoving JL, Assendelft WJJ, Roland M, van Tulder MW. Antidepressants for non-specific low back pain. Cochrane Database Syst Rev. 2008(1):CD001703. 10.1002/14651858.CD001703.pub3 PMC7025781 18253994

[33] Abdel Shaheed C, Maher CG, Williams KA, Day R, McLachlan AJ. Efficacy, tolerability, and dose-dependent effects of opioid analgesics for low back pain: a systematic review and meta-analysis. JAMA Int Med. 2016;176(7):958–68. 10.1001/jamainternmed.2016.1251 27213267

[34] Seidel S, Aigner M, Ossege M, Pernicka E, Wildner B, Sycha T. Antipsychotics for acute and chronic pain in adults. Cochrane Database Syst Rev. 2013(8):CD004844. 10.1002/14651858.CD004844.pub3 PMC10575602 23990266

[35] Tucker H-R, Scaff K, McCloud T, Carlomagno K, Daly K, Garcia A, et al. Harms and benefits of opioids for management of non-surgical acute and chronic low back pain: a systematic review. Br J Sports Med. 2020;54:664. 10.1136/bjsports-2018-099805 30902816

[36] Bagg MK, McLachlan AJ, Maher CG, Kamper SJ, Williams CM, Henschke N, et al. Paracetamol, NSAIDS and opioid analgesics for chronic low back pain: a network meta-analysis. Cochrane Database Syst Rev. 2018(6):CD013045.

[37] Bagg MK, Salanti G, McAuley JH (2018). Comparing interventions with network meta-analysis. J Physiother..

[38] Cipriani A, Higgins JP, Geddes JR, Salanti G (2013). Conceptual and technical challenges in network meta-analysis. Ann Intern Med..

[39] Salanti G. Indirect and mixed-treatment comparison, network, or multiple-treatments meta-analysis: many names, many benefits, many concerns for the next generation evidence synthesis tool. Res Synth Methods. 2012;3(2):80–97. 10.1002/jrsm.1037 26062083

[40] Moher D, Shamseer L, Clarke M, Ghersi D, Liberati A, Petticrew M (2015). Preferred reporting items for systematic review and meta-analysis protocols (PRISMA-P) 2015 statement. Syst Rev..

[41] Shamseer L, Moher D, Clarke M, Ghersi D, Liberati A, Petticrew M, et al. Preferred reporting items for systematic review and meta-analysis protocols (PRISMA-P) 2015: elaboration and explanation. BMJ. 2015;349:g7647. 10.1136/bmj.g7647 25555855

[42] Bagg MK, O’Hagan E, Zahara P, Wand BM, Hübscher M, Moseley GL, et al. Reviews may overestimate the effectiveness of medicines for back pain: systematic review and meta-analysis. J Clin Epidemiol. 2019;124:149–59. 10.1016/j.jclinepi.2019.12.006 31816418

[43] Turner EH, Matthews AM, Linardatos E, Tell RA, Rosenthal R. Selective publication of antidepressant trials and its influence on apparent efficacy. N Engl J Med. 2008;358(3):252–60. 10.1056/NEJMsa065779 18199864

[44] Spineli LM, Higgins JP, Cipriani A, Leucht S, Salanti G (2013). Evaluating the impact of imputations for missing participant outcome data in a network meta-analysis. Clin Trials..

[45] Elbourne DR, Altman DG, Higgins JP, Curtin F, Worthington HV, Vail A (2002). Meta-analyses involving cross-over trials: methodological issues. Int J Epidemiol..

[46] Cipriani A, Furukawa TA, Salanti G, Chaimani A, Atkinson LZ, Ogawa Y, et al. Comparative efficacy and acceptability of 21 antidepressant drugs for the acute treatment of adults with major depressive disorder: a systematic review and network meta-analysis. Lancet. 2018;391(10128):1357–66. 10.1016/S0140-6736(17)32802-7 PMC5889788 29477251

[47] Furukawa TA, Salanti G, Atkinson LZ, Leucht S, Ruhe HG, Turner EH (2016). Comparative efficacy and acceptability of first-generation and second-generation antidepressants in the acute treatment of major depression: protocol for a network meta-analysis. BMJ Open..

[48] Furlan A, Chaparro LE, Irvin E, Mailis-Gagnon A (2011). A comparison between enriched and nonenriched enrollment randomized withdrawal trials of opioids for chronic noncancer pain. Pain Res Manag..

[49] Yamato TP, Maher CG, Saragiotto BT, Shaheed CA, Moseley AM, Lin C-WC, et al. Comparison of effect sizes between enriched and nonenriched trials of analgesics for chronic musculoskeletal pain: a systematic review. Br J Clin Pharmacol. 2017;83(11):2347–55. 10.1111/bcp.13350 PMC5651314 28636752

[50] Koes BW, van Tulder MW, Peul WC (2007). Diagnosis and treatment of sciatica. BMJ..

[51] PDR. Prescriber’s Digital Reference. Available from https://www.pdr.net.

[52] MIMS. MIMS. Available from https://www.mims.co.uk.

[53] AMH. Australian Medicines Handbook. Available from https://amhonline.amh.net.au.

[54] Higgins JPT, Thomas J, Chandler J, Cumpston M, Li T, Page MJ, et al. Cochrane Handbook for Systematic Reviews of Interventions Version 6.0 (updated July 2019). Cochrane; 2011. 10.1002/14651858.ED000142 PMC10284251 31643080

[55] US Food and Drug Administration. Code of Federal Regulations Title 21. 2018.

[56] Tomlinson A, Efthimiou O, Boaden K, New E, Mather S, Salanti G, et al. Side effect profile and comparative tolerability of 21 antidepressants in the acute treatment of major depression in adults: protocol for a network meta-analysis. Evid Based Ment Health. 2019;22(2):61–6. 10.1136/ebmental-2019-300087 PMC10270374 30996028

[57] Innovation VH. Covidence systematic review software. Melbourne, Australia: Veritas Health Innovation. Available at www.covidence.org.

[58] Liberati A, Altman DG, Tetzlaff J, Mulrow C, Gotzsche PC, Ioannidis JP (2009). The PRISMA statement for reporting systematic reviews and meta-analyses of studies that evaluate healthcare interventions: explanation and elaboration. BMJ..

[59] Wan X, Wang W, Liu J, Tong T. Estimating the sample mean and standard deviation from the sample size, median, range and/or interquartile range. BMC Med Res Methodol. 2014;14:135. 10.1186/1471-2288-14-135 PMC4383202 25524443

[60] R Core Team. R: A language and environment for statistical computing. Vienna: R Foundation for Statistical Computing; 2019.

[61] Hjermstad MJ, Fayers PM, Haugen DF, Caraceni A, Hanks GW, Loge JH, et al. Studies comparing Numerical Rating Scales, Verbal Rating Scales, and Visual Analogue Scales for assessment of pain intensity in adults: a systematic literature review. J Pain Symptom Manage. 2011;41(6):1073–93. 10.1016/j.jpainsymman.2010.08.016 21621130

[62] Smeets R, Köke A, Lin C-W, Ferreira M, Demoulin C. Measures of function in low back pain/disorders: Low Back Pain Rating Scale (LBPRS), Oswestry Disability Index (ODI), Progressive Isoinertial Lifting Evaluation (PILE), Quebec Back Pain Disability Scale (QBPDS), and Roland-Morris Disability Questionnaire (RDQ). Arthritis Care Res (Hoboken). 2011;63(S11):S158–S73. 10.1002/acr.20542 22588742

[63] Borenstein M, Hedges LV, Higgins JP, Rothstein HR. Introduction to Meta-Analysis. Chichester: Wiley; 2009.

[64] Borenstein M, Higgins JPT, Hedges LV, Rothstein HR. Basics of meta-analysis: I^2^ is not an absolute measure of heterogeneity. Res Synth Methods. 2017;8(1):5–18. 10.1002/jrsm.1230 28058794

[65] Jansen JP, Naci H. Is network meta-analysis as valid as standard pairwise meta-analysis? It all depends on the distribution of effect modifiers. BMC Med. 2013;11(1):159. 10.1186/1741-7015-11-159 PMC3707819 23826681

[66] Saragiotto BT, Maher CG, Moseley AM, Yamato TP, Koes BW, Sun X, et al. A systematic review reveals that the credibility of subgroup claims in low back pain trials was low. J Clin Epidemiol. 2016;79:3–9. 10.1016/j.jclinepi.2016.06.003 27297201

[67] Dechartres A, Trinquart L, Faber T, Ravaud P. Empirical evaluation of which trial characteristics are associated with treatment effect estimates. J Clin Epidemiol. 2016;77:24–37. 10.1016/j.jclinepi.2016.04.005 27140444

[68] Donegan S, Dias S, Tudur-Smith C, Marinho V, Welton NJ. Graphs of study contributions and covariate distributions for network meta-regression. Res Synth Methods. 2018;9(2):243–60. 10.1002/jrsm.1292 PMC6001528 29377598

[69] Rücker G, Krahn U, König J, Efthimiou O, Schwarzer G. netmeta: Network meta-analysis using frequentist methods. R package version 1.1-0. 2019. https://CRAN.R-project.org/package=netmeta.

[70] Rücker G. Network meta-analysis, electrical networks and graph theory. Res Synth Methods. 2012;3(4):312–24. 10.1002/jrsm.1058 26053424

[71] Rücker G, Schwarzer G. Ranking treatments in frequentist network meta-analysis works without resampling methods. BMC Med Res Methodol. 2015;15(1):58. 10.1186/s12874-015-0060-8 PMC4521472 26227148

[72] Salanti G, Del Giovane C, Chaimani A, Caldwell DM, Higgins JPT. Evaluating the quality of evidence from a network meta-analysis. PLoS One. 2014;9(7):e99682. 10.1371/journal.pone.0099682 PMC4084629 24992266

[73] Schwarzer G, Carpenter JR, Rücker G, Schwarzer G, Carpenter JR, Rücker G (2015). Network Meta-Analysis. Meta-Analysis with R.

[74] Higgins JPT, Whitehead A. Borrowing strength from external trials in a meta-analysis. Stat Med. 1996;15(24):2733–49. 10.1002/(SICI)1097-0258(19961230)15:24<2733::AID-SIM562>3.0.CO;2-0 8981683

[75] Higgins JPT, Jackson D, Barrett JK, Lu G, Ades AE, White IR. Consistency and inconsistency in network meta-analysis: concepts and models for multi-arm studies. Res Synth Methods. 2012;3(2):98–110. 10.1002/jrsm.1044 PMC4433772 26062084

[76] White IR, Barrett JK, Jackson D, Higgins JPT. Consistency and inconsistency in network meta-analysis: model estimation using multivariate meta-regression. Res Synth Methods. 2012;3(2):111–25. 10.1002/jrsm.1045 PMC4433771 26062085

[77] Dias S, Welton NJ, Caldwell DM, Ades AE. Checking consistency in mixed treatment comparison meta-analysis. Stat Med. 2010;29(7-8):932–44. 10.1002/sim.3767 20213715

[78] Veroniki AA, Vasiliadis HS, Higgins JPT, Salanti G (2013). Evaluation of inconsistency in networks of interventions. Int J Epidemiol..

[79] van Valkenhoef G, Kuiper J. Gemtc: Network Meta-Analysis Using Bayesian Methods; 2016. https://cran.r-project.org/web/packages/gemtc/index.html.

[80] van Valkenhoef G, Lu G, de Brock B, Hillege H, Ades AE, Welton NJ. Automating network meta-analysis. Res Synth Methods. 2012;3(4):285–99. 10.1002/jrsm.1054 26053422

[81] Huhn M, Nikolakopoulou A, Schneider-Thoma J, Krause M, Samara M, Peter N, et al. Comparative efficacy and tolerability of 32 oral antipsychotics for the acute treatment of adults with multi-episode schizophrenia: a systematic review and network meta-analysis. Lancet. 2019;394(10202):939–51. 10.1016/S0140-6736(19)31135-3 PMC6891890 31303314

[82] Chaimani A, Salanti G. Using network meta-analysis to evaluate the existence of small-study effects in a network of interventions. Res Synth Methods. 2012;3(2):161–76. 10.1002/jrsm.57 26062088

[83] Efthimiou O, Debray TPA, van Valkenhoef G, Trelle S, Panayidou K, Moons KGM, et al. GetReal in network meta-analysis: a review of the methodology. Res Synth Methods. 2016;7(3):236–63. 10.1002/jrsm.1195 26754852

[84] Donegan S, Welton NJ, Tudur Smith C, D'Alessandro U, Dias S. Network meta-analysis including treatment by covariate interactions: consistency can vary across covariate values. Res Synth Methods. 2017;8(4):485–95. 10.1002/jrsm.1257 PMC5724666 28732142

[85] Donegan S, Dias S, Welton NJ. Assessing the consistency assumptions underlying network meta-regression using aggregate data. Res Synth Methods. 2019;10(2):207–24. 10.1002/jrsm.1327 PMC6563470 30367548

[86] Jansen JP, Schmid CH, Salanti G. Directed acyclic graphs can help understand bias in indirect and mixed treatment comparisons. J Clin Epidemiol. 2012;65(7):798–807. 10.1016/j.jclinepi.2012.01.002 22521579

[87] Peters JL, Sutton AJ, Jones DR, Abrams KR, Rushton L. Contour-enhanced meta-analysis funnel plots help distinguish publication bias from other causes of asymmetry. J Clin Epidemiol. 2008;61(10):991–6. 10.1016/j.jclinepi.2007.11.010 18538991

[88] ISPM. CINeMA: confidence in network meta-analysis. cinema.ispm.unibe.ch: Institute of Social and Preventive Medicine, University of Bern; 2017.

[89] Nikolakopoulou A, Higgins JPT, Papakonstantinou T, Chaimani A, Del Giovane C, Egger M, et al. CINeMA: An approach for assessing confidence in the results of a network meta-analysis. PLoS Med. 2020:17(4):e1003082. 10.1371/journal.pmed.1003082 PMC7122720 32243458

[90] Brignardello-Petersen R, Bonner A, Alexander PE, Siemieniuk RA, Furukawa TA, Rochwerg B, et al. Advances in the GRADE approach to rate the certainty in estimates from a network meta-analysis. J Clin Epidemiol. 2018;93:36–44. 10.1016/j.jclinepi.2017.10.005 29051107

[91] Yepes-Nuñez JJ, Li S-A, Guyatt G, Jack SM, Brozek JL, Beyene J, et al. Development of the summary of findings table for network meta-analysis. J Clin Epidemiol. 2019;115:1–13. 10.1016/j.jclinepi.2019.04.018 31055177

