# Supplementary material for: Analgesic medicines for adults with low back pain: protocol for a systematic review and network meta-analysis
Source: Syst Rev. 2020 Nov 4;9:255. doi: 10.1186/s13643-020-01506-3 (PMC7643321; doi:10.1186/s13643-020-01506-3)
Supplement: Supplementary file 2 — Additional file 2. Interventions of Interest. [file 13643_2020_1506_MOESM2_ESM.docx]

**Additional File 2. Interventions of Interest**

| **Type** | **Name** | **ATC code** | **Notes** |
| --- | --- | --- | --- |
| Opioids | morphine | N02AA01 |  |
|  | morphine combinations | N02AA51 |  |
|  | hydromorphone | N02AA03 |  |
|  | oxymorphone | N02AA |  |
|  | nicomorphine | N02AA04 |  |
|  | oxycodone | N02AA05 |  |
|  | oxycodone and naloxone | N02AA55 |  |
|  | oxycodone and paracetamol | N02AJ17 |  |
|  | oxycodone and acetylsalicylic acid | N02AJ18 |  |
|  | oxycodone and ibuprofen | N02AJ19 |  |
|  | codeine | R05DA04 |  |
|  | codeine, combinations excl. psycholeptics | N02AA59 | where these are NSAIDs, as defined herein, or Paracetamol |
|  | codeine and paracetamol | N02AJ06 |  |
|  | codeine and acetylsalicylic acid | N02AJ07 |  |
|  | codeine and ibuprofen | N02AJ08 |  |
|  | codeine and other non-opioid analgesics | N02AJ09 | where these are NSAIDs, as defined herein, or Paracetamol |
|  | dihydrocodeine | N02AA08 |  |
|  | dihydrocodeine combinations | N02AA58 | where these are combinations with NSAIDs, as defined herein, or Paracetamol |
|  | dihydrocodeine and paracetamol | N02AJ01 |  |
|  | dihydrocodeine and acetylsalicylic acid | N02AJ02 |  |
|  | dihydrocodeine and other non-opioid analgesics | N02AJ03 | where these are NSAIDs, as defined herein, or Paracetamol |
|  | papaveretum | N02AA10 |  |
|  | buprenorphine | N02AE01 |  |
|  | tilidine | N02AX01 |  |
|  | dezocine | N02AX03 |  |
|  | meptazinol | N02AX05 |  |
|  | tapentadol | N02AX06 |  |
|  | tramadol | N02AX02 |  |
|  | tramadol and paracetamol | N02AJ13 |  |
|  | tramadol and dexketoprofen | N02AJ14 |  |
|  | tramadol and other non-opioid analgesics | N02AJ15 | where these are NSAIDs, as defined herein, or Paracetamol |
|  | butaphornal | N02AF01 |  |
|  | nalbuphine | N02AF02 |  |
|  | ketobemidone | N02AB01 |  |
|  | pethidine | N02AB02 |  |
|  | pethidine, combinations excl. psycholeptics | N02AB52 | where these are NSAIDs, as defined herein, or Paracetamol |
|  | fentanyl | N02AB03 |  |
|  | dextromoromide | N02AC01 |  |
|  | piritramide | N02AC03 |  |
|  | dextropropoxyphene | N02AC04 |  |
|  | bezitramide | N02AC05 |  |
|  | methadone | N07BC02 |  |
|  | methadone, combinations excl. psycholeptics | N02AC52 | where these are NSAIDs, as defined herein, or Paracetamol |
|  | dextropropoxyphene, combinations excl.  psycholeptics | N02AC54 | where these are NSAIDs, as defined herein, or Paracetamol |
|  | pentazocine | N02AD01 |  |
|  | phenazocine | N02AD02 |  |
| NSAIDs | phenylbutazone | M01AA01 |  |
|  | mofebutazone | M01AA02 |  |
|  | oxyphenbutazone | M01AA03 |  |
|  | piroxicam | M01AC01 |  |
|  | lornoxicam | M01AC02 |  |
|  | meloxicam | M01AC06 |  |
|  | ibuprofen | M01AE01 |  |
|  | naproxen | M01AE02 |  |
|  | ketoprofen | M01AE03 |  |
|  | fenoprofen | M01AE04 |  |
|  | flurbiprofen | M01AE09 |  |
|  | tiaprofenic acid | M01AE11 |  |
|  | oxaprozin | M01AE12 |  |
|  | dexibruprofen | M01AE14 |  |
|  | dexketoprofen | M01AE17 |  |
|  | mefenamic acid | M01AG01 |  |
|  | tolfenamic acid | M01AG02 |  |
|  | meclofenamic acid | M01AG04 |  |
|  | indomethacin | M01AB01 |  |
|  | sulindac | M01AB02 |  |
|  | tolmetin | M01AB03 |  |
|  | zomepirac | M01AB04 |  |
|  | diclofenac | M01AB05 |  |
|  | alclofenac | M01AB06 |  |
|  | etodolac | M01AB08 |  |
|  | ketorolac | M01AB15 |  |
|  | aceclofenac | M01AB16 |  |
|  | bufexamac | M01AB17 |  |
|  | celecoxib | M01AH01 |  |
|  | valdecoxib | M01AH03 |  |
|  | parecoxib | M01AH04 |  |
|  | etoricoxib | M01AH05 |  |
|  | nabumetone | M01AX01 |  |
|  | glucosamine | M01AX05 |  |
|  | GAG polysulfate | M01AX12 |  |
|  | nimesulide | M01AX17 |  |
|  | diflunisal | N02BA11 |  |
|  | chondroitin sulphate | M01AX25 |  |
| Paracetamol | paracetamol (acetaminophen) | N02BE01 |  |
|  | acetylsalicylic acid (aspirin) | N02BA01 |  |
|  | acetylsalicylic acid, combinations excl. psycholeptics | M01BA03 | where these are NSAIDs, as defined herein, or Paracetamol |
| Muscle relaxants | Suxamethonium | M03AB01 |  |
|  | botulinum toxin | M03AX01 |  |
|  | Pancuronium | M03AC01 |  |
|  | Vecuronium | M03AC03 |  |
|  | Atracurium | M03AC04 |  |
|  | rocuronium bromide | M03AC09 |  |
|  | mivacurium bromide | M03AC10 |  |
|  | Cisatracurium | M03AC11 |  |
|  | Carisoprodol | M03BA02 |  |
|  | Methocarbamol | M03BA03 |  |
|  | Chlorzoxazone | M03BB03 |  |
|  | orphenadrine citrate | M03BC01 |  |
|  | baclofen | M03BX01 |  |
|  | tizanidine | M03BX02 |  |
|  | tolperisone | M03BX04 |  |
|  | thiocolchicoside | M03BX05 |  |
|  | cyclobenzaprine | M03BX08 |  |
|  | dantrolene | M03CA01 |  |
|  | clonazepam | N03AE01 |  |
|  | diazepam | N05BA01 |  |
|  | chlordiazepoxide | N05BA02 |  |
|  | oxazepam | N05BA04 |  |
|  | lorazepam | N05BA06 |  |
|  | bromazepam | N05BA08 |  |
|  | clobazam | N05BA09 |  |
|  | alprazolam | N05BA12 |  |
|  | clotiazepam | N05BA21 |  |
|  | flurazepam | N05CD01 |  |
|  | nitrazepam | N05CD02 |  |
|  | flunitrazepam | N05CD03 |  |
|  | estazolam | N05CD04 |  |
|  | triazolam | N05CD05 |  |
|  | lormetazepam | N05CD06 |  |
|  | temazepam | N05CD07 |  |
|  | midazolam | N05CD08 |  |
|  | quazepam | N05CD10 |  |
|  | zolpidem | N05CF02 |  |
|  | zaleplon | N05CF03 |  |
|  | eszopiclone | N05CF04 |  |
|  | metaxalone | - |  |
| Anticonvulsants | Methylphenobarbital | N03AA01 |  |
|  | Phenobarbital | N03AA01 |  |
|  | Primidone | N03AA03 |  |
|  | Ethotoin | N03AB01 |  |
|  | phenytoin | N03AB02 |  |
|  | Fosphenytoin | N03AB05 |  |
|  | ethosuximide | N03AD01 |  |
|  | mesuximide | N03AD03 |  |
|  | Clonazepam | N03AE01 |  |
|  | carbamazepine | N03AF01 |  |
|  | oxcarbazepine | N03AF02 |  |
|  | Rufinamide | N03AF03 |  |
|  | eslicarbazepine | N03AF04 |  |
|  | valproic acid | N03AG01 |  |
|  | Valpromide | N03AG02 |  |
|  | vigabatrin | N03AG04 |  |
|  | Tiagabine | N03AG06 |  |
|  | Sultiame | N03AX03 |  |
|  | lamotrigine | N03AX09 |  |
|  | felbamate | N03AX10 |  |
|  | topiramate | N03AX11 |  |
|  | gabapentin | N03AX12 |  |
|  | levetiracetam | N03AX14 |  |
|  | zonisamide | N03AX15 |  |
|  | pregabalin | N03AX16 |  |
|  | stiripentol | N03AX17 |  |
|  | lacosamide | N03AX18 |  |
|  | perampanel | N03AX22 |  |
|  | brivaracetam | N03AX23 |  |
| Antidepressants | agomelatine | N06AX22 |  |
|  | amineptine | N06AA19 |  |
|  | amitriptyline | N06AA09 |  |
|  | amoxapine | N06AA17 |  |
|  | bupropion | N06AX12 |  |
|  | butriptyline | N06AA15 |  |
|  | citalopram | N06AB04 |  |
|  | clomipramine | N06AA04 |  |
|  | desipramine | N06AA01 |  |
|  | desvenlafaxine | N06AX23 |  |
|  | dibenzepin | N06AA08 |  |
|  | dimetacrine | N06AA18 |  |
|  | dosulepin | N06AA16 |  |
|  | doxepin | N06AA12 |  |
|  | duloxetine | N06AX21 |  |
|  | escitalopram | N06AB10 |  |
|  | etoperidone | N06AB09 |  |
|  | fluoxetine | N06AB03 |  |
|  | fluvoxamine | N06AB08 |  |
|  | gepirone | N06AX19 |  |
|  | imipramine | N06AA02 |  |
|  | imipramine oxide | N06AA03 |  |
|  | isocarboxazid | N06AF01 |  |
|  | levomilnacipran | - |  |
|  | maprotiline | N06AA21 |  |
|  | mianserin | N06AX03 |  |
|  | milnacipran | N06AX17 |  |
|  | mirtazapine | N06AX11 |  |
|  | moclobemide | N06AG02 |  |
|  | nefazodone | N06AX06 |  |
|  | nortriptyline | N06AA10 |  |
|  | oxitriptan | N06AX01 |  |
|  | paroxetine | N06AB05 |  |
|  | phenelzine | N06AF03 |  |
|  | protriptyline | N06AA11 |  |
|  | reboxetine | N06AX18 |  |
|  | sertraline | N06AB06 |  |
|  | tianeptine | N06AX14 |  |
|  | tranylcypromine | N06AF04 |  |
|  | trazodone | N06AX05 |  |
|  | trimipramine | N06AA06 |  |
|  | tryptophan | N06AX02 |  |
|  | venlafaxine | N06AX16 |  |
|  | vilazodone | N06AX24 |  |
|  | vortioxetine | N06AX26 |  |
| Corticosteroids | Fludrocortisone | H02AA02 |  |
|  | betamethasone | H02AB01 |  |
|  | dexamethasone | H02AB02 |  |
|  | methylprednisolone | H02AB04 |  |
|  | prednisolone | H02AB06 |  |
|  | Prednisone | H02AB07 |  |
|  | triamcinolone | H02AB08 |  |
|  | hydrocortisone | H02AB09 |  |
|  | Cortisone | H02AB10 |  |
|  | rimexolone | H02AB12 |  |
|  | deflazacort | H02AB13 |  |
