# Supplementary material for: Analgesic medicines for adults with low back pain: protocol for a systematic review and network meta-analysis
Source: Syst Rev. 2020 Nov 4;9:255. doi: 10.1186/s13643-020-01506-3 (PMC7643321; doi:10.1186/s13643-020-01506-3)
Supplement: Supplementary file 3 — Additional file 3. MEDLINE search strategies. [file 13643_2020_1506_MOESM3_ESM.docx]

**Additional File 3: Search Strategy for MEDLINE (OVID):**

***Part A: Generic search for randomized controlled trials***

1. randomized controlled trial.pt.

2. controlled clinical trial.pt.

3. comparative study.pt.

4. clinical trial.pt.

5. random*.ab.

6. placebo.ab,ti.

7. drug therapy.fs.

8. trial.ab,ti.

9. groups.ab,ti.

10. or/1-9

11. (animals not (humans and animals)).sh.

12. (adolescent* or teen* or youth? or puberty or childhood or children* or p?ediatri* or preschool or pre-school or nursery or kindergarten or infant? or newborn? or neonat* or prematurity or fetal or foetal).mp.

13. 11 or 12

14. 10 not 13

***Part B: Specific search for low back, sacrum and coccyx problems***

15. dorsalgia.ti,ab.

16. exp Back Pain/

17. backache.ti,ab.

18. ((lumb* or back) adj pain).ti,ab.

19. coccydynia.ti,ab.

20. sciatica.ti,ab.

21. spondylosis.ti,ab.

22. lumbago.ti,ab.

23. back disorder$.ti,ab

24. or/15-23

***Part C: Specific search for other spinal disorders***

25. Coccyx.sh

26. Lumbar Vertebrae.sh

27. Intervertebral disc.sh

28. discitis.ti,ab.

29. Sacrum.sh

30. Intervertebral disc degeneration.sh

31. (disc adj degenerat*).ti,ab.

32. (disc adj prolapse*).ti,ab.

33. (disc adj herniat*).ti,ab.

34. spinal fusion.sh.

35. (facet adj joint*).ti,ab.

36. Intervertebral Disc Displacement.sh.

37. postlaminectomy.ti,ab.

38. or/25-37

***Part D: Specific search for interventions of interest***

39. morphine.sh or morphine.mp

40. hydromorphone.sh or hydromorphone.mp

41. oxycodone.sh or oxycodone.mp

42. oxymorphone.sh or oxymorphone.mp

43. nicomorphine.mp

44. codeine.sh or codeine.mp

45. exp naloxone/ or naloxone.mp

46. dihydrocodeine.mp

47. papaveratum.mp

48. buprenorphine.sh or buprenorphine.mp

49. tilidine.sh or tilidine.mp

50. dezocine.mp

51. meptazinol.sh or meptazinol.mp

52. tapentadol.mp

53. tramadol.sh or tramadol.mp

54. butaphornal.mp

55. nalbuphine.sh or nalbuphine.mp

56. ketobemidone.mp

57. meperidine.sh or meperidine.mp or pethidine.mp

58. fentanyl.sh or fentanyl.mp

59. dextromoramide.sh or dextromoramide.mp

60. piritramide.sh or piritramide.mp

61. dextropropoxyphene.sh or dextropropoxyphene.mp

62. bezitramide.mp

63. methadone.sh or methadone.mp

64. pentazocine.sh or pentazocine.mp

65. phenazocine.sh or phenazocine.mp

66. phenylbutazone.sh or phenylbutazone.mp

67. mofebutazone.mp

68. oxyphenbutazone.sh or oxyphenbutazone.mp

69. meloxicam.mp

70. piroxicam.sh or piroxicam.mp

71. lornoxicam.mp

72. ibuprofen.sh or ibuprofen.mp

73. naproxen.sh or naproxen.mp

74. ketoprofen.sh or ketoprofen.mp

75. fenoprofen.sh or fenoprofen.mp

76. flurbiprofen.sh or flurbiprofen.mp

77. tiaprofenic acid.mp

78. oxaprozin.mp

79. dexibuprofen.mp

80. dexketoprofen.mp

81. mefenamic acid.sh or mefenamic acid.mp

82. tolfenamic acid.mp

83. meclofenamic acid.sh or meclofenamic acid.mp

84. exp indomethacin/ or indomethacin.mp or indometacin.mp

85. sulindac.sh or sulindac.mp

86. tolmetin.sh or tolmetin.mp

87. zomepirac.mp

88. diclofenac.sh or diclofenac.mp

89. alclofenac.mp

90. etodolac.sh or etodolac.mp

91. aceclofenac.mp

92. bufexamac.sh or bufexamac.mp

93. celecoxib.sh or celecoxib.mp

94. valdecoxib.mp

95. etoricoxib.mp

96. nabumetone.mp

97. exp glucosamine/ or glucosamine.mp

98. glucosaminoglycan polysulfate.mp

99. nimesulide.mp

100. chondroitin sulfate.mp

101. diflunisal.sh or diflunisal.mp

102. acetaminophen.sh or acetaminophen.mp or paracetamol.mp

103. aspirin.sh or aspirin.mp or acetylsalicylic acid.mp

104. cebranopadol.mp

105. hydrocodone.sh or hydrocodone.mp

106. ketorolac.sh or ketorolac.mp

107. analgesics.sh

108. analgesics, non-narcotic.sh

109. analgesics, opioid.sh

110. analgesics, short-acting.sh

111. prostaglandin antagonists.sh

112. anti-inflammatory agents, non-steroidal.sh

113. cyclooxygenase inhibitors.sh

114. cyclooxygenase 2 inhibitors.sh

115. suxamethonium.mp or succinylcholine.sh or succinylcholine.mp

116. exp botulinum toxins/ or botulinum toxin.mp

117. pancuronium.sh or pancuronium.mp

118. vecuronium bromide.sh or vecuronium bromide.mp

119. atracurium.sh or atracurium.mp

120. rocuronium.sh or rocuronium.mp

121. mivacurium bromide.mp

122. cisatracurium.mp.

123. carisoprodol.sh or carisoprodol.mp

124. methocarbamol.sh or methocarbamol.mp

125. chlorzoxazone.sh or chlorzoxazone.mp

126. orphenadrine.sh or orphenadrine.mp

127. baclofen.sh or baclofen.mp

128. tizanidine.mp.

129. tolperisone.sh or tolperisone.mp

130. thiocolchicoside.mp.

131. cyclobenzaprine.mp.

132. dantrolene.sh or dantrolene.mp

133. clonazepam.sh or clonazepam.mp

134. exp Diazepam/ or diazepam.mp

135. chlordiazepoxide.sh or chlordiazepoxide.mp

136. oxazepam.sh or oxazepam.mp

137. lorazepam.sh or lorazepam.mp

138. bromazepam.sh or bromazepam.mp

139. clobazam.sh or clobazam.mp

140. alprazolam.sh or alprazolam.mp

141. clotiazepam.mp.

142. flurazepam.sh or flurazepam.mp

143. nitrazepam.sh or nitrazepam.mp

144. flunitrazepam.sh or flunitrazepam.mp

145. estazolam.sh or estazolam.mp

146. triazolam.sh or triazolam.mp

147. lormetazepam.mp.

148. temazepam.sh or temazepam.mp

149. midazolam.sh or midazolam.mp

150. quazepam.mp.

151. zolpidem.sh or zolpidem.mp

152. zaleplon.mp.

153. eszopiclone.sh or eszopiclone.mp

154. agomelatine.mp

155. amineptine.mp

156. amitriptyline.sh or amitriptyline.mp

157. amoxapine.sh or amoxapine.mp

158. bupropion.sh or bupropion.mp or buproprion.mp

159. butriptyline.mp

160. citalopram.sh or citalopram.mp or escitalopram.mp

161. clomipramine.sh or clomipramine.mp

162. desipramine.sh or desipramine.mp

163. desvenlafaxine succinate.sh or desvenlafaxine.mp

164. dibenzepin.sh or dibenzepin.mp

165. dimetacrine.mp

166. dothiepin.sh or dosulepin.mp

167. doxepin.sh or doxepin.mp

168. duloxetine hydrochloride.sh or duloxetine.mp

169. etoperidone.mp

170. fluoxetine.sh or fluoxetine.mp

171. fluvoxamine.sh or fluvoxamine.mp

172. gepirone.mp

173. imipramine.sh or imipramine.mp or imipramine oxide.mp

174. isocarboxazid.sh or isocarboxazid.mp

175. levomilnacipran.mp

176. maprotiline.sh or maprotiline.mp

177. mianserin.sh or mianserin.mp

178. exp Milnacipran/ or milnacipran.mp

179. mirtazapine.sh or mirtazapine.mp

180. moclobemide.sh or moclobemide.mp

181. nefazodone.mp

182. nortriptyline.sh or nortriptyline.mp

183. oxitriptan.mp

184. paroxetine.sh or paroxetine.mp

185. phenelzine.sh or phenelzine.mp

186. protriptyline.sh or protriptyline.mp

187. reboxetine.sh or reboxetine.mp

188. sertraline.sh or sertraline.mp

189. tianeptine.mp

190. tranylcypromine.sh or tranylcypromine.mp

191. trazodone.sh or trazodone.mp

192. trimipramine.sh or trimipramine.mp

193. exp Tryptophan/ or tryptophan.mp

194. venlafaxine hydrochloride.sh or venlafaxine.mp

195. vilazodone hydrochloride.sh or vilazodone.mp

196. vortioxetine.sh or vortioxetine.mp

197. mephobarbital.sh or methylphenobarbital.mp

198. exp Phenobarbital/ or phenobarbital.mp

199. primidone.mp

200. ethotoin.mp

201. phenytoin.sh or phenytoin.mp

202. fosphenytoin.mp

203. ethosuximide.sh or ethosuximide.mp
204. mesuximide.mp

205. exp carbamazepine/ or carbamazepine.mp

206. oxcarbazepine.mp

207. rufinamide.mp

208. eslicarbazepine.mp

209. valproic acid.sh or valproic acid.mp

210. valpromide.mp

211. vigabatrin.sh or vigabatrin.mp

212. tiagabine.sh or tiagabine.mp

213. sultiame.mp or sulthiame.mp

214. lamotrigine.sh or lamotrigine.mp

215. felbamate.sh or felbamate.mp

216. topiramate.sh or topiramate.mp

217. gabapentin.sh or gabapentin.mp

218. levetiracetam.sh or levetiracetam.mp

219. zonisamide.sh or zonisamide.mp

220. pregabalin.sh or pregabalin.mp

221. stiripentol.mp

222. lacosamide.sh or lacosamide.mp

223. perampanel.mp

224. brivaracetam.mp

225. cannabidiol.sh or cannabidiol.mp

226. fludrocortisone.mp

227. exp Betamethasone/ or betamethasone.mp

228. exp Dexamethasone/ or dexamethasone.mp

229. prednisone.sh or prednisone.mp

230. exp Prednisolone/ or prednisolone.mp or methylprednisolone.mp

231. exp Triamcinolone/ or triamcinolone.mp

232. exp Hydrocortisone/ or hydrocortisone.mp

233. cortisone.sh or cortisone.mp

234. rimexolone.mp

235. deflazacort.mp

236. metaxalone.mp

237. exp Desoxycorticosterone/ or desoxycorticosterone.mp

238. exp Fluocortolone or fluocortolone.mp

239. or/39-238 (all interventions of interest)

***Results***

240. 24 or 38 (all back pain)

241. 239 and 240 (all back pain and all interventions of interest)

242. 14 and 241 (all RCTs of interventions of interest in back pain)
