# Supplementary material for: Analgesic medicines for adults with low back pain: protocol for a systematic review and network meta-analysis
Source: Syst Rev. 2020 Nov 4;9:255. doi: 10.1186/s13643-020-01506-3 (PMC7643321; doi:10.1186/s13643-020-01506-3)
Supplement: Supplementary file 4 — Additional file 4. CINeMA. [file 13643_2020_1506_MOESM4_ESM.docx]

**Additional File 4: Process for forming judgements of confidence**

***Judgment on confidence in the pairwise comparisons***

Within-study bias: We will evaluate within-study bias using the approach outlined in the Risk of bias section. We will rate each direct comparison as at low, moderate or high risk of bias, assigning scores of 0, −1 and −2, respectively (1). We will consider direct comparisons at high risk of bias when > 25% of participants in the comparison are from studies at high overall risk of bias, low risk when > 50% of participants are from studies at low overall risk and moderate risk in all other instances. We will use the contributions of all direct comparisons to each pairwise effect (from the contribution matrix) to construct weighted averages of these scores. Then we will base judgements of the confidence to be placed in each pairwise effect on a weighted average of the risk of bias of direct comparisons feeding into it. We will downgrade one level for −1.5 < score < −0.5 and two levels for scores < −1.51, although this may change in practice if there is clear imbalance in the contribution of evidence that would render such action inappropriate.

Reporting bias: Our systematic review includes a comprehensive literature search for published and unpublished data, which reduces the risk of reporting (publication) bias. Nevertheless, we will evaluate reporting bias for each outcome with contour-enhanced funnel plots for all analgesic medicines vs placebo (2). If we judge funnel plot asymmetry, we shall downgrade all comparisons by one level, except in the following situation: where 10 or more studies for a specific comparison are available, in which case a contour-enhanced funnel plot will be produced for this comparison only (3). We will re-examine the new funnel plot and, if we judge no evidence of asymmetry, we will not downgrade the respective comparison.

Indirectness: Salanti *et al.* propose indirectness be considered with the transitivity assumption (4). We assume that, based on our inclusion criteria, there will be no indirectness and studies will fulfil the assumption of transitivity. Given the lack of known effect modifiers for LBP (5), we will consider five potential effect modifiers during our assessment of transitivity (see *Assumption of transitivity*). We will consider downgrading a pairwise effect one level for indirectness if direct comparisons with important imbalances in populations, treatments, or outcomes contribute the majority of information to it. In addition, we will downgrade all comparisons one additional level if there were concerns over the transitivity assumption identified prior to the analysis.

Imprecision: We will evaluate imprecision by considering the width of the 95% CI for pairwise effects. We will downgrade one level when they span either the null or the threshold for a clinically meaningful effect on pain intensity (10 points on a 0 to 100 scale (6)) and two levels when the interval spans both. We will not consider sample sizes as there are no established criteria for this evaluation in the NMA context (4).

Heterogeneity: We will evaluate each direct comparison for heterogeneity/consistency in the direction and magnitude of the effect sizes from individual trials, considering the width of the prediction interval and magnitude of the heterogeneity parameter. We will downgrade direct comparisons one level if important heterogeneity is identified – intervals spanning greater than 15 points (on a 0 to 100 scale).

Incoherence: We will downgrade one level any direct comparisons that are implicated in loops with important incoherence or where there is a discrepancy between direct and indirect evidence.

***Judgment of confidence in the treatment rankings***

Within-study bias: We will use the judgements of overall risk of bias for each direct comparison (see above) and construct a weighted average for the entire network using the percentage contributions of each direct comparison to the entire network. We will downgrade one level for −1.5 < score < −0.5 and two levels for scores < −1.51, although this may change in practice if there is clear imbalance in the contribution of evidence that would render such action inappropriate.

Reporting bias: We will evaluate the likelihood of reporting (publication) bias across the network, using the evidence of reporting bias from direct comparisons (see above) and the comparison-adjusted funnel plots. We acknowledge that plot asymmetry may occur to factors other than reporting bias (4). We will downgrade one level if there is evidence of publication bias or small study effects.

Indirectness: We will use our judgements of indirectness within pairwise comparisons (see above) and evaluate the contribution to the entire network by comparisons that are judged to exhibit indirectness. We will consider downgrading one level if there is an important contribution from one/more of these comparisons. In addition, we will downgrade all comparisons one additional level if there were concerns over the transitivity assumption identified prior to the analysis.

Imprecision: We will evaluate the precision of the treatment rankings by examining the P-scores that are used to calculate these probabilities. We will deem rankings imprecise when there are similar probabilities for two/more treatment to be ranked at the same level and will downgrade one level in this situation.

Heterogeneity/incoherence: We will consider the magnitude of the heterogeneity parameter from the NMA model and the result of the Chi² test for global incoherence, acknowledging that we may fail to detect important global incoherence due to the low power of such a test and the presence of large heterogeneity (7-9). We will downgrade one level if either heterogeneity or incoherence are present and two levels if both are present.

**References**

1. Bagg MK, McLachlan AJ, Maher CG, Kamper SJ, Williams CM, Henschke N, et al. Paracetamol, NSAIDS and opioid analgesics for chronic low back pain: a network meta‐analysis. Cochrane Database of Systematic Reviews. 2018(6).

2. Peters JL, Sutton AJ, Jones DR, Abrams KR, Rushton L. Contour-enhanced meta-analysis funnel plots help distinguish publication bias from other causes of asymmetry. Journal of clinical epidemiology. 2008;61(10):991-6.

3. Huhn M, Nikolakopoulou A, Schneider-Thoma J, Krause M, Samara M, Peter N, et al. Comparative efficacy and tolerability of 32 oral antipsychotics for the acute treatment of adults with multi-episode schizophrenia: a systematic review and network meta-analysis. The Lancet. 2019;394(10202):939-51.

4. Salanti G, Del Giovane C, Chaimani A, Caldwell DM, Higgins JPT. Evaluating the Quality of Evidence from a Network Meta-Analysis. PLOS ONE. 2014;9(7):e99682.

5. Saragiotto BT, Maher CG, Moseley AM, Yamato TP, Koes BW, Sun X, et al. A systematic review reveals that the credibility of subgroup claims in low back pain trials was low. Journal of clinical epidemiology. 2016;79:3-9.

6. Chou R, Deyo R, Friedly J, Skelly A, Weimer M, Fu R, et al. Systemic Pharmacologic Therapies for Low Back Pain: A Systematic Review for an American College of Physicians Clinical Practice GuidelineSystemic Pharmacologic Therapies for Low Back Pain. Annals of internal medicine. 2017;166(7):480-92.

7. Higgins JPT, Jackson D, Barrett JK, Lu G, Ades AE, White IR. Consistency and inconsistency in network meta-analysis: concepts and models for multi-arm studies. Research synthesis methods. 2012;3(2):98-110.

8. Veroniki AA, Mavridis D, Higgins JPT, Salanti G. Characteristics of a loop of evidence that affect detection and estimation of inconsistency: a simulation study. BMC medical research methodology. 2014;14:106-.

9. Veroniki AA, Vasiliadis HS, Higgins JPT, Salanti G. Evaluation of inconsistency in networks of interventions. International Journal of Epidemiology. 2013;42(1):332-45.
